# 

**Published:** 1866-10

**Authors:** 


### CONTENTS.

ORIGINAL COMMUNICATIONS.

Dental Materia Medica ... 424
Food and the Teeth ... 428

PROCEEDINGS OF SOCIETIES.

Discussions of Mississippi Valley Dental Association ... 439

EDITORIAL.

Nitrous Oxide as an Anæsthetic ... 467
Dental Education ... 469
Crystal Shred Gold ... 469
Instructions in Nitrous Oxide ... 470
Books ... 470

